# Melatonin ameliorates restraint stress-induced oxidative stress and apoptosis in testicular cells via NF-κB/iNOS and Nrf2/ HO-1 signaling pathway

**DOI:** 10.1038/s41598-017-09943-2

**Published:** 2017-08-29

**Authors:** Ying Guo, Junyan Sun, Ting Li, Qiuwan Zhang, Shixia Bu, Qian Wang, Dongmei Lai

**Affiliations:** International Peace Maternity and Child Health Hospital, School of Medicine, Shanghai Jiao Tong University, Shanghai, 200030 China

## Abstract

Decline in semen quality has become a global public health concern. Psychological stress is common in the current modern society and is associated with semen decline. Increasing evidence demonstrated that melatonin has anti-apoptotic and antioxidant functions. Whether melatonin can ameliorate the damage in testes induced by psychological stress has never been investigated. Here, a mouse model of restraint stress demonstrated that melatonin normalized the sperm density decline, testicular cells apoptosis, and testicular oxidative stress in stressed male mice. Melatonin decreased reactive oxygen species (ROS) level, increased superoxide dismutase (SOD) and glutathione (GSH) activities, and downregulated inducible nitric oxide synthase (iNOS) and tumor necrosis factor-α (TNF-α) activities in stressed mice testes. Furthermore, melatonin reduced the stress-induced activation of the NF-κB signaling pathway by decreasing the phosphorylation of nuclear factor of kappa light polypeptide gene enhancer in B-cells inhibitor, alpha (IκBα) and p65 nuclear translocation. In addition, melatonin upregulated the expression of anti-oxidant proteins including nuclear factor erythroid 2-related factor 2 (Nrf2) and heme oxygenase-1 (HO-1). Meanwhile, *in vitro* studies also demonstrated melatonin could reduce oxidative apoptosis of testicular cells. Collectively, melatonin mitigated psychological stress-induced spermatogenic damage, which provides evidence for melatonin as a therapy against sperm impairment associated with psychological stress.

## Introduction

Approximately 10–15% of couples are infertile worldwide, and the male counterpart affects 40–60% of infertility^1, 2^. Semen quality is a well-recognized marker of fertility and a sentinel indicator of gamete deterioration. Several studies suggest a decline in semen quality around the world^3–9^, which has become a global public health concern. Although the potential causes for the decline have not yet been determined, psychological stress has been reported to be associated with decreased quality of semen^10–14^.

Psychological stress is common in the current modern society^15^. A recent survey indicated that 25% of the population in the USA reported high stress and 50% identified a major stressful event during the previous year^15^. Restraint stress is widely utilized to mimic the psychological stress in many studies, in which the individual is isolated from its group and his movement is confined to a restricted area^16, 17^. Increasing evidence demonstrates that chronic stress, including restraint stress, results in a significant decline in the quality of semen^18–20^, which could be related to a disruption in testosterone secretion in testes^20–22^, and an increase of oxidative stress^20, 23, 24^, and apoptosis of germ cells^19, 24, 25^.

Melatonin is mainly secreted by the pineal gland and is one of the most well-investigated antioxidants; it can scavenge a variety of free radicals^26–29^. Melatonin also upregulates the expression of antioxidant proteins^27, 30, 31^ and defends against oxidants-induced damage in many tissues^30, 32–34^. Oxidative stress is one of the major factors that induce testicular cells apoptosis in testes^35–37^. Several studies showed that stress induced alterations in spermatogenesis potentially due to the increased oxidative stress in testes^20, 23, 24^. In addition, some other studies demonstrated that melatonin has an anti-apoptotic effect in somatic and germ cells^22, 38–40^. Melatonin has been reported to be protective in male reproductive health, which readily crosses the blood-testis barrier and has a very low toxicity^41, 42^. Studies have investigated the use of melatonin to relieve the side effects of chemotherapy drugs and environmental toxins during spermatogenesis^22, 43–46^. However, few systematic studies have investigated whether melatonin exerts a protective role in the psychological stress-induced impairment of spermatogenesis as well as the mechanisms by which melatonin mitigates the damage in testes.

Therefore, in this study, we used a mouse model of restraint stress in order to investigate the effects of melatonin on stress-induced testicular cells apoptosis and oxidative stress and explored the mechanisms underlying the beneficial effects of melatonin.

## Results

### Effects of melatonin on chronic restraint stress-induced body weight loss, disturbed serum corticosterone level and melatonin content in serum and testes tissues of mice

**Figure 1:**
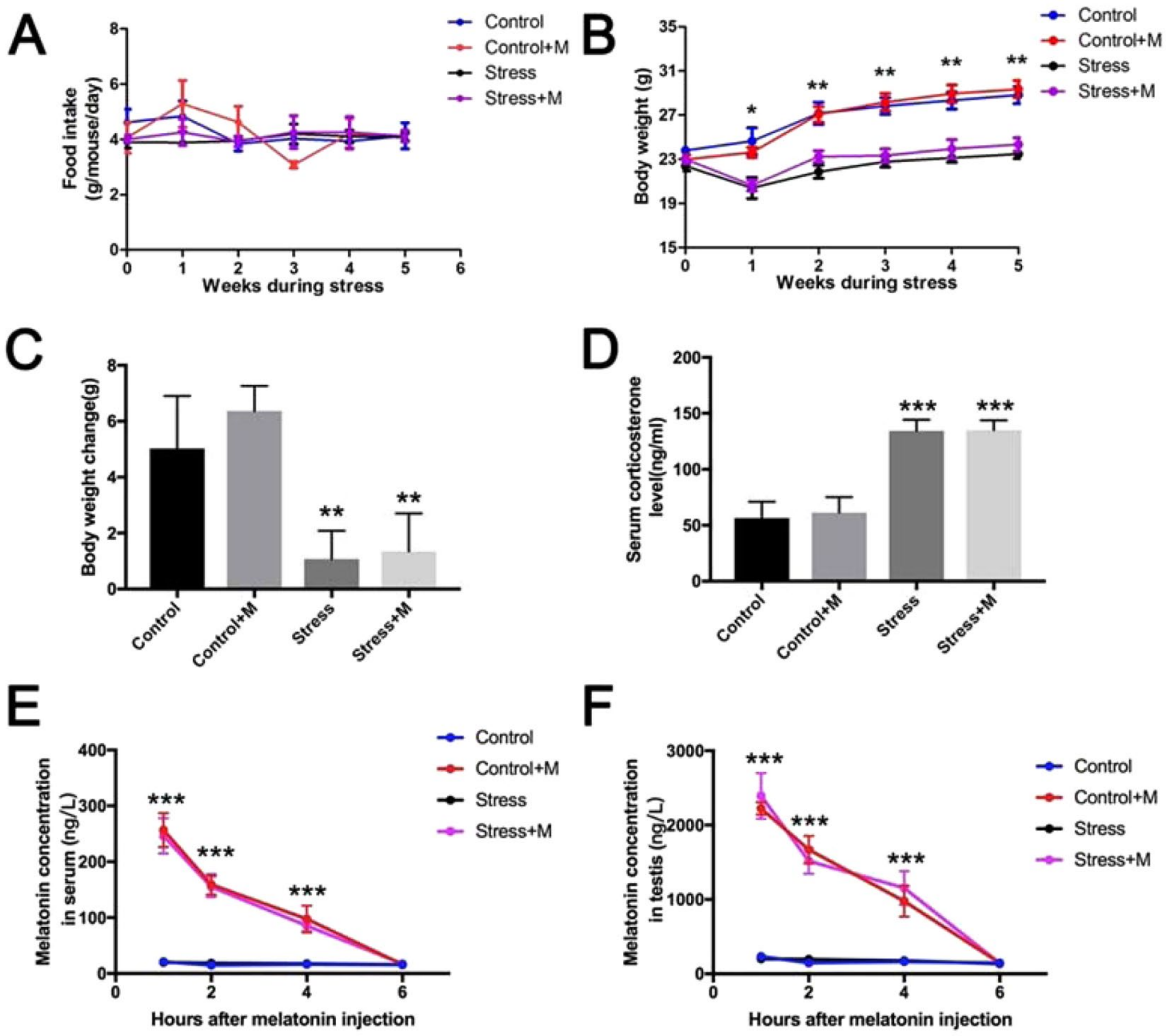
Effects of melatonin (M) on the stress status of restraint stress mice and the content of melatonin in mice. (**A**) Food intake of mice in four groups (n = 10). (**B**) Body weight trajectories (**C**) Body weight change of each group of mice (n = 10). (**D**) Serum corticosterone level in mice (n = 6). (**E**) Serum melatonin level in mice (n = 6). (**F**) Melatonin level in testes of mice (n = 6). Data are presented as mean ± SEM. *P < 0.05 *vs*. control group; **P < 0.01 *vs*. control group; ***P < 0.001 *vs*. control group. Control, non-stress treated with saline; Control+M, non-stress treated with melatonin; Stress, stress treated with saline; Stress+M, stress treated with melatonin.

In the present study, mouse restraint system was utilized for the physical immobilization-induced chronic stress. Briefly, the chronic stress was applied using a 50 mL conical centrifuge tube for 6 h/day with multiple punctures for sufficient ventilation. During the 35 days of stress, the body weight and food intake of mice in different groups were monitored once every week. A significant difference was not observed in the food intake in either of the groups (P > 0.05, n = 10) (Fig. 1A). Body weight trajectories and changes of every group were illustrated (Fig. 1B,C). Compared to the control group, the stressed mice presented a reduced body weight gain (5.0 ± 0.8 g *vs*. 1.1 ± 0.5 g, P < 0.05, n = 10) (Fig. 1C); whereas melatonin treatment (10 mg/kg/day) did not relieve the altered body weight compared to that in stressed mice (1.1 ± 0.5 g *vs*. 1.3 ± 0.6 g, P > 0.05, n = 10) (Fig. 1C). Consistently, exposure to melatonin of control mice also did not influence the body weight compared to that of control mice (6.3 ± 0.4 g *vs*. 5.0 ± 0.8 g, P > 0.05, n = 10) (Fig. 1C). In order to evaluate the stress level in different groups, serum corticosterone concentration was estimated after restraint stress for 5 weeks. Results showed that the corticosterone level in stressed mice was significantly higher than that in the control mice (134.2 ± 4.5 ng/ml *vs*. 56.4 ± 6.6 ng/ml, P < 0.001, n = 6), and it was comparable between the stressed mice and melatonin-treated stressed mice (134.2 ± 4.5 ng/ml *vs*. 135.0 ± 4.0 ng/ml, P > 0.05, n = 6) (Fig. 1D), with comparable level between the control mice and melatonin-treated control mice (61.22 ± 6.209 ng/ml *vs*. 56.4 ± 6.6 ng/ml, P > 0.05, n = 6) (Fig. 1D). In addition, the concentrations of melatonin in serum and testes in melatonin-treated group were significantly increased 1 h, 2 h and 4 h after injection (Fig. 1E,F, n = 6 and Supplementary Table 1). However, the concentrations of melatonin in serum and testes in melatonin-treated group were comparable with that in untreated mice 6 h after injection (Fig. 1E,F, n = 6). Of note, stress condition posed no effects on the concentration of melatonin in blood and testes (Fig. 1E,F, n = 6 and Supplementary Table 1).

### Effects of melatonin on restraint stress-induced spermatogenesis impairment

**Figure 2:**
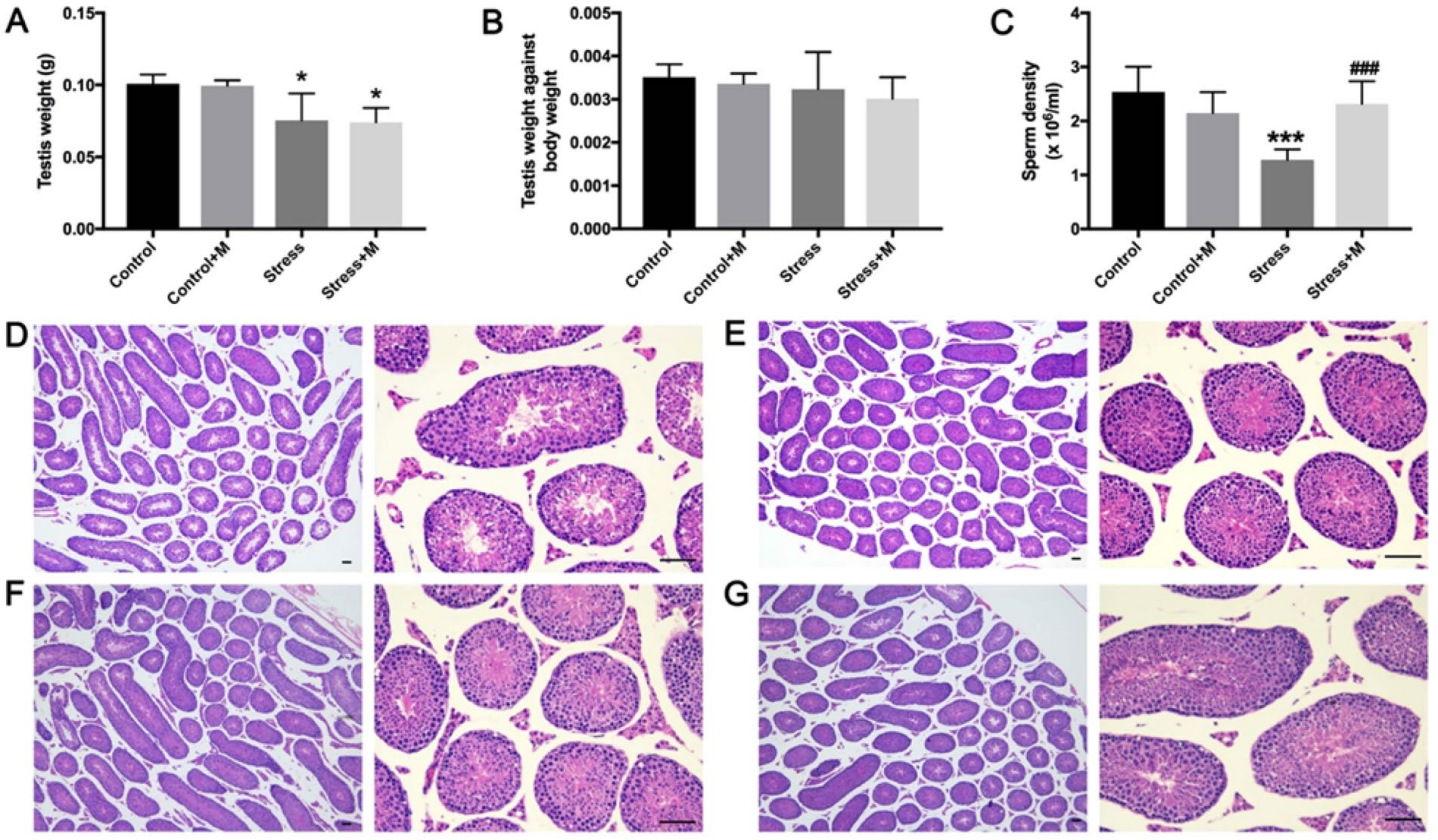
Effects of melatonin (M) on restraint stress-induced spermatogenesis impairment. (**A**) Absolute testes weight (n = 6). (**B**) The relative weight of testes against the body weight (n = 6). (**C**) Sperm density of mice in different groups (n = 6). (**D–G**) Histology of testes in control mice, control+M, stress, and stress+M group, respectively (Bar = 100 μm). All data are presented as mean ± SEM. *P < 0.05 *vs*. control group; ***P < 0.001 *vs*. control group; ^###^P < 0.001 *vs*. stress group. Control, non-stress treated with saline; Control+M, non-stress treated with melatonin; Stress, stress treated with saline; Stress+M, stress treated with melatonin.

To evaluate the spermatogenesis impairment in mice testes, the testes weight, sperm density, and histology of the testes were examined. The absolute testes weight was decreased in both stressed (0.0756 ± 0.008 g *vs*. 0.1010 ± 0.003 g, P < 0.05, n = 6) and melatonin-treated stressed mice (0.0742 ± 0.004 g *vs*. 0.1010 ± 0.003 g, P < 0.05, n = 6) as compared to the controls (Fig. 2A), and testes weight was comparable between melatonin-treated control mice and control group (0.0995 ± 0.002 g *vs*. 0.1010 ± 0.003 g, P > 0.05, n = 6). However, the relative weight of testes against body weight did not exhibit significant differences among each group (P > 0.05, n = 6) (Fig. 2B). As expected, the spermatogenesis output namely the sperm density was notably decreased in stressed mice (1.280 ± 0.087 × 10^6^/ml *vs*. 2.538 ± 0.191 × 10^6^/ml, P < 0.001, n = 6) (Fig. 2C). Interestingly, melatonin almost completely alleviated the stress-induced reduction in sperm count (2.317 ± 0.170 × 10^6^/ml *vs*. 1.280 ± 0.087 × 10^6^/ml, P < 0.001, n = 6) (Fig. 2C), and the sperm density was not influenced by melatonin when compared that of melatonin-treated mice to the control mice (2.150 ± 0.157 × 10^6^/ml *vs*. 2.538 ± 0.191 × 10^6^/ml) (Fig. 2C). However, the histopathological evaluation of testes in different groups was indistinguishable by naked eyes (Fig. 2D–G).

### Melatonin treatment inhibits the restraint stress-induced testicular cells apoptosis and the activation apoptotic cascade in testes

**Figure 3:**
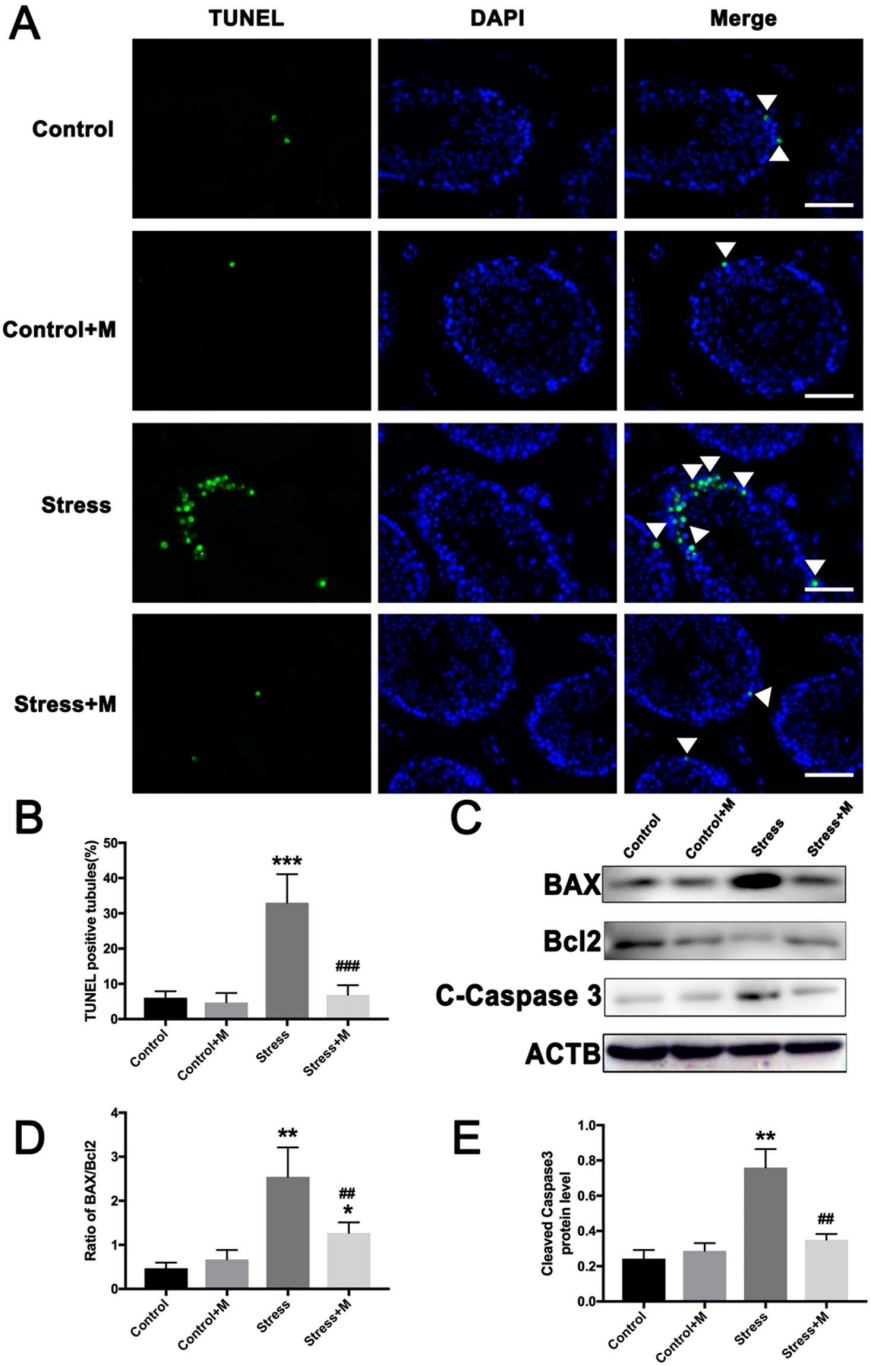
Effects of melatonin (M) on chronic stress-induced apoptosis of testicular cells and apoptotic cascade in testes. (**A**) Testicular cells apoptosis was detected by TUNEL staining (Bar = 100 μm). The arrow shows apoptotic testicular cells in seminiferous tubules. (**B**) Percentages of seminiferous tubules with more than six TUNEL+ cells (n = 5). (**C**) The protein levels of BAX, Bcl2, and cleaved caspase 3 were detected by Western blot (n = 5). (**D**) The ratio of pro-apoptotic protein BAX over anti-apoptotic protein Bcl-2 (n = 5). (**E**) Cleaved caspase 3 protein level was analyzed (n = 5). ACTB protein level served as the internal reference. All data are presented as mean ± SEM. *P < 0.05 *vs*. control group; **P < 0.01 *vs*. control group; ***P < 0.001 *vs*. control group; ^#^P < 0.05 *vs*. stress group; ^##^P < 0.01 *vs*. stress group. Control, non-stress treated with saline; Control+M, non-stress treated with melatonin; Stress, stress treated with saline; Stress+M, stress treated with melatonin.

The decline in semen density potentially caused by apoptosis of testicular cells was investigated based on the apoptosis of testicular cells by TUNEL. As shown in Fig. 3A,B, testicular cells apoptosis was significantly enhanced in the stressed group with an increased proportion of tubules with more than 6 TUNEL+ cells (33.03 ± 4.662% *vs*. 6.056 ± 1.056%, P < 0.001, n = 5). Strikingly, melatonin significantly ameliorated the apoptosis of testicular cells induced by chronic stress (6.905 ± 1.561% *vs*. 33.03 ± 4.662%, P < 0.001, n = 5) (Fig. 3A,B). DNase I treatment was used as a positive control and the staining solution was used as a negative control (see Supplementary Fig. S1A–B). Next, the apoptotic pathway was analyzed in testes which contains all testicular cells. Compared to controls, the ratio of pro-apoptotic protein BAX over anti-apoptotic protein Bcl-2 was significantly augmented in stressed male testes (Fig. 3C,D, n = 5). Accordingly, the activity of cleaved caspase-3 was also significantly increased (Fig. 3C and E, n = 5). Consistently, melatonin markedly normalized the ratio of BAX over Bcl-2 (Fig. 3C,D, n = 5) and decreased the level of cleaved caspase-3 (Fig. 3C and E, n = 5) in the testes of stressed mice.

### Melatonin treatment improves the restraint stress-induced oxidative stress in testicular cells

**Figure 4:**
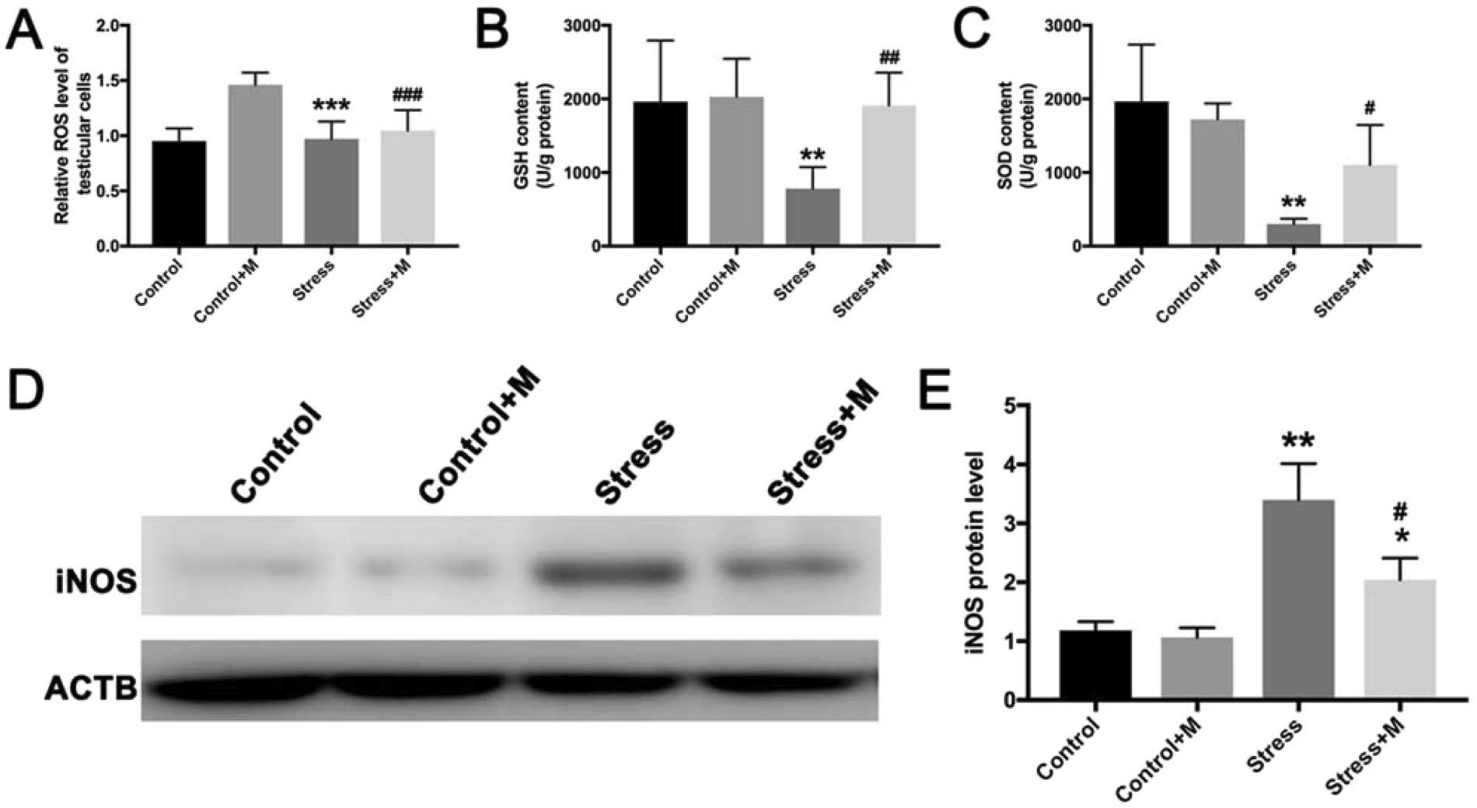
Effects of melatonin (M) on chronic stress-induced oxidative stress in testes. (**A**) ROS level relative to control group (n = 6). (**B**) GSH content in different groups (n = 6). (**C**) SOD content in different groups (n = 6). (**D–E**) The protein level of iNOS detected by Western blot, and relative level compared to the housekeeping molecule ACTB (n = 5). All data are presented as mean ± SEM. *P < 0.05 *vs*. control group; **P < 0.01 *vs*. control group; ***P < 0.001 *vs*. control group; ^#^P < 0.05 *vs*. stress group; ^##^P < 0.01 *vs*. stress group; ^###^P < 0.001 *vs*. stress group. Control, non-stress treated with saline; Control+M, non-stress treated with melatonin; Stress, stress treated with saline; Stress+M, stress treated with melatonin.

Given that the oxidative stress is a major contributor to restraint stress-induced spermatogenic damage that could lead to testicular cells apoptosis^24, 26, 27^, we next investigated the status of oxidative stress in the testes of each group and also, the level of ROS was evaluated. As shown in Fig. 4A, the level of ROS increased approximately 1.5-fold in the stressed group as compared to that in control group (Fig. 4A, n = 6). The antioxidant molecules, such as SOD (298.8 ± 35.8 U/g protein *vs*. 1965.0 ± 386.6 U/g protein, P < 0.01, n = 6) and GSH (782.3 ± 118.8 μg/g protein *vs*. 1960.0 ± 339.7 μg/g protein, P < 0.01, n = 6), were markedly decreased in the stressed group (Fig. 4B,C). In addition, stressed testes exhibited significantly elevated levels of the pro-oxidant enzyme, iNOS (Fig. 4D–E, n = 5). Conversely, melatonin significantly normalized the levels of total ROS, SOD (1102.0 ± 271.4 U/g protein *vs*. 298.8 ± 35.8 U/g protein, P < 0.05, n = 6), GSH (1912.1 ± 180.8 μg/g protein *vs*. 782.3 ± 118.8 μg/g protein, P < 0.01, n = 6), and iNOS (Fig. 4A–D).

### Melatonin treatment reduces the restraint stress-induced activation of NF-κB signaling pathway and normalizes Nrf2/HO-1 signaling pathway

**Figure 5:**
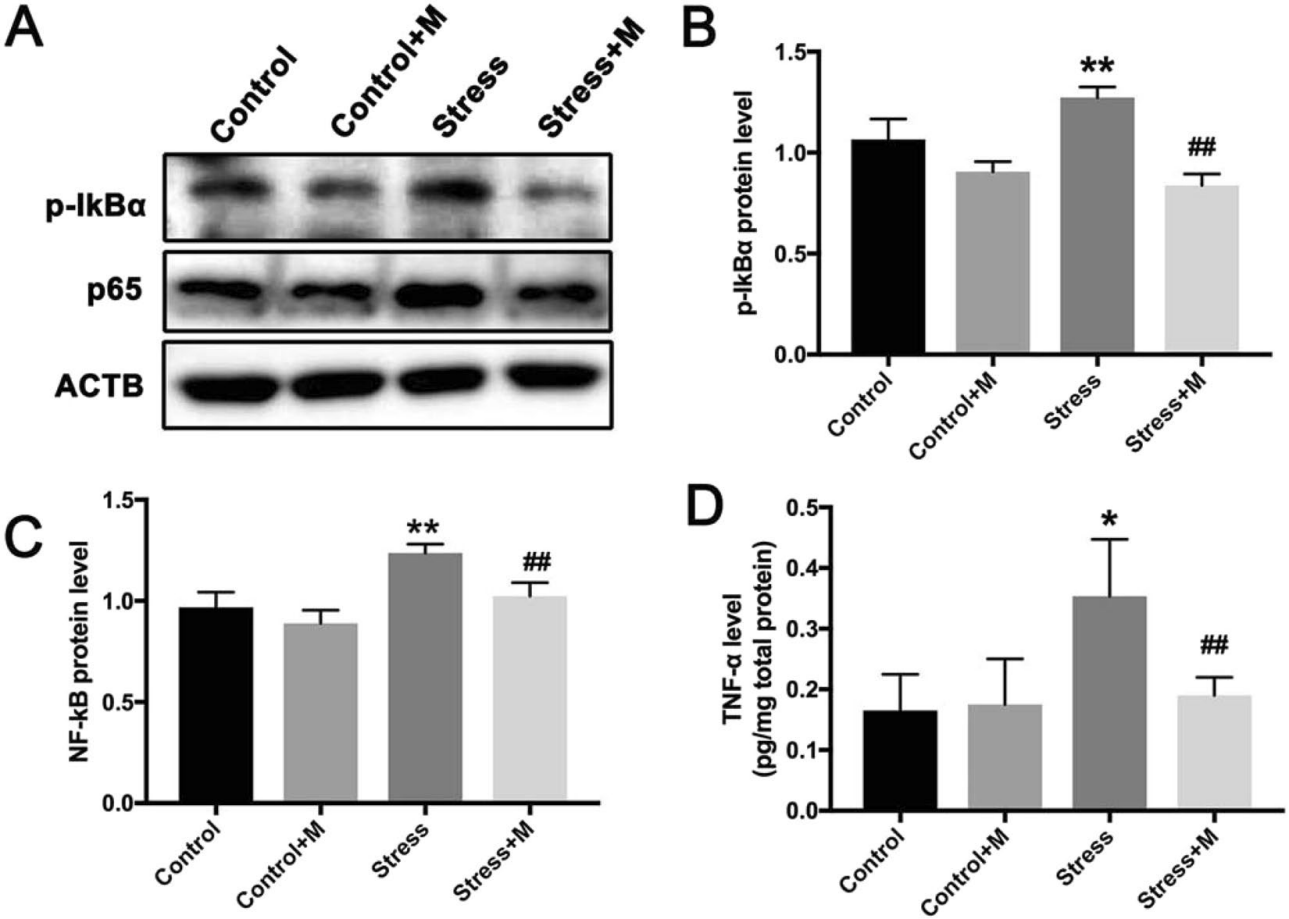
Effects of melatonin (M) on chronic stress-induced activation of NF-κB signaling pathway in testes. (**A**) The expression of p-IκBα and nuclear protein level of p65 were measured by Western Blot (n = 5). (**B**) The relative expression level of p-IκBα against ACTB (n = 5). (**C**) The nuclear protein level of p65 against ACTB (n = 5). (**D**) The level of TNF-α in testes detected by ELISA (n = 5). All data are presented as mean ± SEM. *P < 0.05 *vs*. control group; **P < 0.01 *vs*. control group; ^##^P < 0.01 *vs*. stress group. Control, non-stress treated with saline; Control+M, non-stress treated with melatonin; Stress, stress treated with saline; Stress+M, stress treated with melatonin.

Oxidative stress can activate the NF-κB signaling pathway that is proapoptotic in testes^47–50^. Thus we examined whether NF-κB signaling pathway in the testes was activated by restraint stress and was associated with the beneficial effects of melatonin. The results showed that the stress notably increased the phosphorylation of IκBα and the nuclear translocation of p65 (Fig. 5A–C, n = 5), while melatonin significantly normalized the level of the two proteins (Fig. 5A–C, n = 5). Since inflammatory cytokines have been found to be induced by chronic stress in testes^49^and also play a vital role in the regulation of iNOS^50^, we further evaluated the level of TNF-α in serum in different group and investigated the effect of melatonin. The level of TNF-α was increased in the stressed group (0.353 ± 0.054 pg/mg protein *vs*. 0.165 ± 0.034 pg/mg protein, P < 0.05, n = 5) that was significantly reduced by melatonin (0.190 ± 0.017 pg/mg protein *vs*. 0.353 ± 0.054 pg/mg protein, P < 0.01, n = 5) (Fig. 5D). But TNF- α content has been not changed in melatonin-treated control group (0.175 ± 0.043 pg/mg protein *vs*. 0.165 ± 0.034 pg/mg protein, P > 0.05, n = 5) (Fig. 5D).

**Figure 6:**
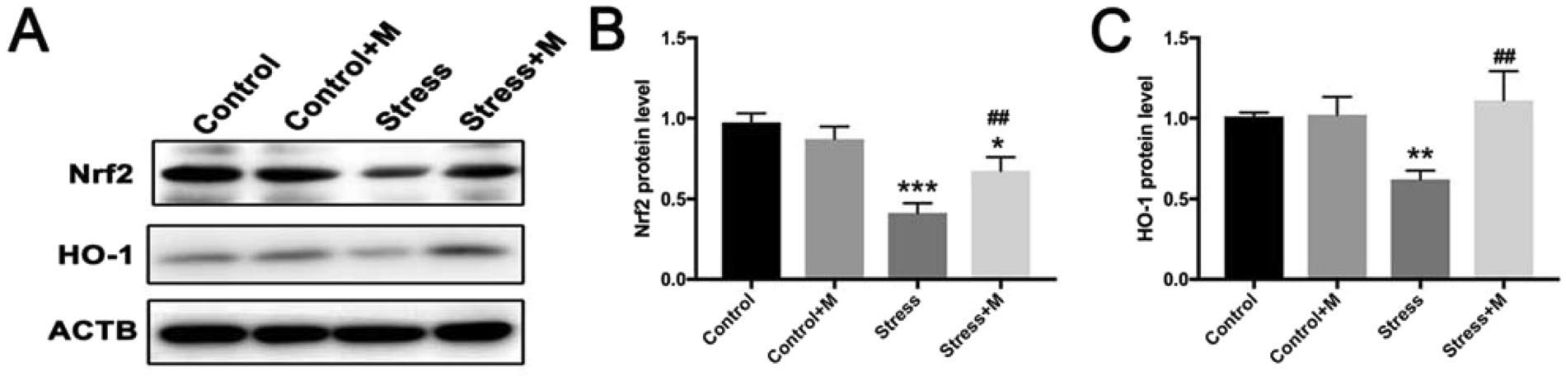
Effects of melatonin (M) and restraint stress on Nrf2/HO-1 signaling pathway. (**A**) The expression of Nrf2 and HO-1 in testes of different groups of mice was detected by Western blot (n = 5). (**B,C**) The relative expression level of Nrf2 and HO-1 normalized to ACTB (n = 5). All data are presented as mean ± SEM. **P < 0.01 *vs*. control group; ***P < 0.001 *vs*. control group; ^##^P < 0.01 *vs*. stress group. Control, non-stress treated with saline; Control+M, non-stress treated with melatonin; Stress, stress treated with saline; Stress+M, stress treated with melatonin.

Nrf2 transcriptionally regulates the antioxidant proteins to maintain redox homeostasis^32^ while HO-1 plays a critical role in the maintenance of cellular homeostasis under stressful conditions^51^. Both molecules act as potent antioxidants. Nrf2 and HO-1 are involved in the heat stress-induced apoptosis of testicular cells^52^. Here, we examined whether the two molecules were associated with restraint stress-induced oxidative stress and whether melatonin exerted a contrasting effect on oxidative stress by regulating Nrf2 and HO-1. As shown in Fig. 6, the stress exposure significantly downregulated the expression of Nrf-2 and HO-1 in mice testes, whereas melatonin significantly alleviated the down-regulated expression (Fig. 6, n = 5).

### Melatonin reduces the H_2_O_2_-induced apoptosis of testicular cells and normalizes NF-κB signaling pathway and Nrf2/HO-1 signaling pathway

**Figure 7:**
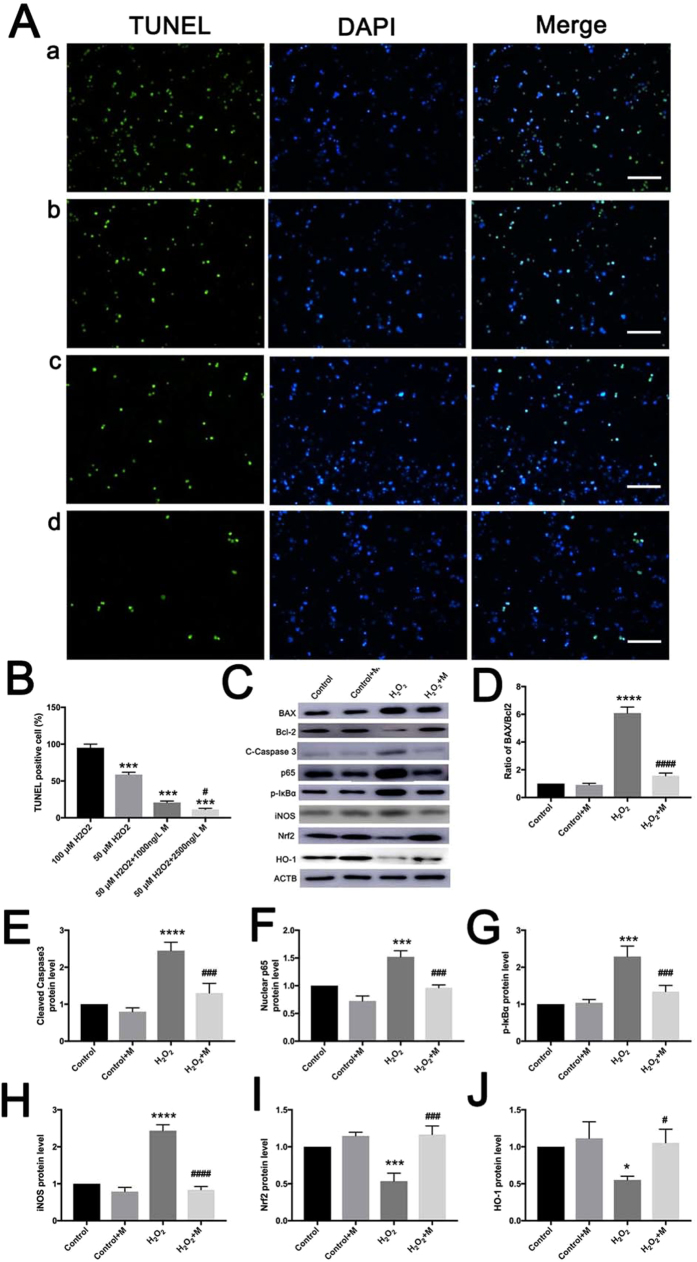
Melatonin reduces the H_2_O_2_-induced apoptosis of testicular cells and activation of NF-κB signaling pathway and Nrf2/HO-1 signaling pathway. (**Aa–Ad**) Testicular cells apoptosis was detected by TUNEL staining in 100 μM H_2_O_2_, 50 μM H_2_O_2_, 1000 ng/L melatonin + 50 μM H_2_O_2_ and 2500 ng/L melatonin + 50 μM H_2_O_2_, respectively (Bar = 100 μm). (**B**) Percentages of TUNEL+ cells (n = 5). (**C**) The expression of BAX, Bcl-2, Cleaved caspases 3, nuclear protein level of p65, p-IκBα, iNOS, Nrf2 and HO-1 were measured by Western Blot. (**D–J**) The relative expression level of BAX/Bcl-2, Cleaved caspases 3, nuclear protein level of p65, p-IκBα, iNOS, Nrf2 and HO-1 normalized to ACTB (n = 5). All data are presented as mean ± SEM. *P < 0.05 *vs*. control group; ***P < 0.001 *vs*. control group; ****P < 0.0001 *vs*. control group; ^#^P < 0.05 *vs*. H_2_O_2_ group; ^###^P < 0.001 *vs*. H_2_O_2_ group; ^####^P < 0.0001 *vs*. H_2_O_2_ group. Control, cultured with normal medium; Control+M, cultured with supplement of 2500 ng/L melatonin; H_2_O_2_, cultured with supplement of 50 μM H_2_O_2_; H_2_O_2_+M, cultured with supplement of 2500 ng/L melatonin and 50 μM H_2_O_2_.

In order to examine the effects of melatonin *in vitro*, testicular cells were treated with H_2_O_2_ to induce oxidative apoptosis, melatonin or both. 100 μM H_2_O_2_ significantly induced the apoptosis of nearly all testicular cells (Fig. 7A-a and B, n = 5), while 50 μM H_2_O_2_ resulted in apoptosis in about 50% testicular cells (Fig. 7A-b and B, n = 5). According to the concentrations detected in testes of mice that were administrated melatonin via intraperitoneal injections, melatonin at 1000 ng/L and 2500 ng/L were chosen to co-treat the testicular cells with H_2_O_2_ *in vitro*. The TUNEL assay results showed melatonin could significantly reduce the apoptosis of testicular cells treated with 50 μM H_2_O_2_ at 1000 ng/L and at 2500 ng/L (Fig. 7A-c,A-d and B, n = 5). In addition, the effects were dose-dependent. DNase I treatment was used as a positive control and the staining solution was used as a negative control (see Supplementary Fig. S2A,B).

Testicular cells were cultured *in vitro* with four conditions: untreated, 2500 ng/L melatonin, 50 μM H_2_O_2_, and both 50 μM H_2_O_2_ and 2500 ng/L melatonin. Accordingly, the ratio of pro-apoptotic protein BAX over anti-apoptotic protein Bcl-2 as well as the level of cleaved caspase-3 were significantly downregulated in melatonin + H_2_O_2_ group (Fig. 7C–E, n = 5). What’s more, melatonin notably decreased the phosphorylation of IκBα (Fig. 7C and G, n = 5) and the nuclear translocation of p65 (Fig. 7C and F, n = 5). Meanwhile, the pro-oxidant protein iNOS was markedly downregulated and antioxidant proteins Nrf2 and HO-1 were significantly upregulated by melatonin (Fig. 7C,H–J, n = 5).

## Discussion

Psychological stress is associated with decreased semen quality in fertile males^10, 12, 13^. In addition, chronic stress induces testicular cells apoptosis in rodents^18–21, 24, 25^. Here, we described a mouse model in which, the animals were exposed to restraint stress for 5 weeks. The reduced body weight gain and the significantly increased serum corticosterone level indicate that the mice underwent the experimental stress. We found that testicular melatonin levels in serum and testes were not affected by restraint stress. Increased melatonin content in both serum and testes tissue after injection indicated melatonin administrated via intraperitoneal injections reached the testes, suggesting that the effects of melatonin are locally and directly exerted in the testes. However, melatonin did not show any effect on the stress status in the stressed mice. Therefore, it was demonstrated that the mice in the stressed and melatonin-treated stressed groups experience a similar stress state. Consistent with previous studies^19, 20, 24^, the restraint stress mice exhibited impairment of spermatogenesis including reduced testicular weight and sperm density. Strikingly, the damage was markedly ameliorated by melatonin. To the best of our knowledge, this is the first report describing that melatonin supplement can improve the stress tolerance of spermatogenesis in mice, which might help manage the negative effects of psychological stress in male reproduction.

Consistent with previous studies^19, 24, 25^, the apoptosis of testicular cells was enhanced by restraint stress in the present study. Several pieces of evidence have demonstrated the anti-apoptotic function of melatonin in tissues such as neural, heart, liver, and kidney^32–34, 38^. In addition, melatonin attenuated the toxin- and drugs-induced apoptotic testicular cells damage in testes^22, 43, 53^. Here, we found that the number of apoptotic testicular cells was significantly reduced when melatonin was supplemented during restraint stress. We also examined the activation of apoptotic pathways in testes. BAX is one of the pro-apoptotic proteins, and Bcl-2 is anti-apoptotic^54^. Cleaved caspase-3 is a crucial effector caspase essential for triggering apoptosis^55^. Herein, the levels of BAX and cleaved caspase-3 were increased in the testes of stressed mice, whereas Bcl-2 was decreased, which confirmed the enhanced apoptosis of testicular cells induced by stress. Furthermore, melatonin reversed the expression of apoptotic proteins. Taken together, these data demonstrated that melatonin could protect against the stress-mediated testicular cells apoptosis.

Stressful conditions lead to excessive production of free radicals that cause an imbalance in the oxidant/antioxidant system^20, 23, 24^, and oxidative stress is one of the major factors that induce testicular cells apoptosis in testes^24, 35, 36^. For instance, chronic stress induces enhanced oxidative stress, oxidative damage, and reduction in the total antioxidant levels in the testes of rats^24^. A previous study found that scrotal heat induced severe oxidative stress in mouse testes, which consequently caused testicular cells death^56^. On the other hand, the antioxidants are well-known for protecting the testicular cells against oxidative damage^35, 57, 58^. Melatonin is a well-established antioxidant exerting a protective role against oxidative stress and apoptosis in somatic^30, 32–34, 38, 40^ and germ cells^22, 43, 59^. However, whether melatonin can protect against restraint stress-mediated oxidative damage of testes is yet unknown. Therefore, we assessed the level of ROS in testes and explored the effects of melatonin. Our results demonstrated that ROS accumulation occurred in the stress group, which was diminished by melatonin treatment. In addition, antioxidants, such as SOD and GSH, were significantly decreased in the stressed mice that recovered almost to baseline level in the melatonin-treated stress group. SOD and GSH constitute the major antioxidant system. SOD is the first line of defense against oxidative stress^60^, and GSH is sensitive to intracellular ROS, which could be neutralized by ROS as one the major endogenous antioxidants^61, 62^. iNOS is one of the primary pro-oxidant enzymes responsible for triggering the cellular injury^63^. However, whether iNOS is regulated by chronic stress is yet to be elucidated. In the present study, we demonstrated that restraint stress reduced the level of SOD and GSH in the testes, thereby suggesting that SOD and GSH were likely attenuated by increased ROS in stressed testes. On the other hand, we also showed that stress treatment increased the expression of iNOS in the testes. Considering the antioxidant functions of melatonin, we further demonstrated that the redox balance was improved by melatonin as evident from the normalized levels of SOD, GSH, and iNOS in the melatonin-treated group. Thus, melatonin may alleviate restraint stress-induced testicular cells apoptosis by relieving the oxidative stress.

Oxidative stress is known to stimulate transcription factors, including NF-κB^47, 51^, and in turn, NF-κB regulates genes, such as iNOS^64^. In addition, NF-κB plays a role in the regulation of testicular cells apoptosis, and it has been determined that the activation of NF-κB is proapoptotic in testicular cells^48^. In this study, we found that NF-κB signaling pathway was activated in the testes by restraint stress, because IκBa, the inhibitor of NF-κB, was phosphorylated remarkably, and the level of nuclear translocation of NF-κB (p65 subunit) increased in the testes of the stress group. NF-κB could be inhibited by melatonin to exert a protective role in diabetic neuropathy and kidney grafts^32, 33^. Herein, we showed that melatonin inhibited the activation of p65 in the testes of the stressed mice as well as in H_2_O_2_-treated testicular cells. In addition, the level of one of the major inflammatory factors, TNF-α, was elevated with the activation of NF-κB that was subsequently abrogated by melatonin. Thus, the stress-induced spermatogenic damage could potentially contribute to NF-κB pathway activation, which is relieved by melatonin.

Given the vital role of ROS in stress condition, it is imperative to address how to limit ROS-induced spermatogenic impairment. Melatonin is known to activate the antioxidant enzymes^32^. Nrf2 plays a significant role in preventing the development of oxidative stress in spermatogenesis^52, 65^. HO-1 has also been reported to be involved in the testicular response to stress^22, 52^. In the current study, we showed that both restraint stress and H_2_O_2_ which could induce oxidative stress significantly reduced the expression of Nrf-2 and HO-1 of testicular cells. Therefore, it can be hypothesized that restraint stress effectuated oxidative stress in the testes by disrupting Nrf2/HO-1 signaling pathway. Several studies found that melatonin is a potent regulator of Nrf2 and HO-1^22, 31, 32^. Melatonin has been shown to modulate neuroinflammation and oxidative stress in experimental diabetic neuropathy via the regulation of NF-κB and Nrf2 pathways^32^. A recent study reported that melatonin activates the Nrf2-ARE pathway when it exhibits a protective effect against early brain injury in a subarachnoid hemorrhage model^31^. Another study indicated that melatonin modulates the expression of Nrf2 to protect against cyclophosphamide-induced urotoxicity^66^. On the other hand, melatonin is demonstrated as a regulator of HO-1^67^, especially, it alleviates the cadmium-induced cellular stress in association with the effect of HO-1^22^. In addition, the ability of melatonin to regulate the Nrf2 pathway is associated with the regulation of HO-1 expression^32, 68^. Moreover, decursin can reduce the oxidative stress induced by Nrf2-mediated upregulation of HO-1 in unilateral cryptorchidism in rat and may improve cryptorchidism-induced infertility^52^. Another group elucidated the protective role of hemin-induced HO-1 on testicular damage caused by acute immobilization stress^69^. Consecutively, in this study, we found that both Nrf2 and HO-1 were significantly enhanced by melatonin in stress-treated testes and H_2_O_2_-treated testicular cells; however, whether HO-1 is directly regulated by melatonin or is dependent on Nrf2 remains unclear. Collectively, these data suggest that the beneficial effect of melatonin are at least partially attributable to the normalization of the expression of Nrf2 and HO-1 in order to attenuate the ROS produced by restraint stress. Testicular cells apoptosis was accompanied by increased ROS in our experiment, which could induce the damage of testicular cells indeed. But whether ROS is the single factor that mediates testicular apoptosis in chronic stress remains unknown. The apoptosis might also be induced by corticosterone as observed during dexamethasone treatment^72^. Therefore, it is possible that both the increase in corticosterone from chronic stressed males and ROS overproduction can induce testicular cells apoptosis by activating apoptotic signaling pathways, which could be ameliorated by melatonin. However, this has yet to be investigated in the future.

In human, the average daytime value of melatonin is 10 ng per liter and peaks at nighttime to 60 ng per liter^73^. Oral doses (1 to 5 mg), which are currently used in the treatment of sleep disorders in humans, result in serum melatonin concentrations that are 10 to 100 times higher within one hour after ingestion, followed by a decline to base-line values in four to eight hours^73^. In the present study, the concentrations of melatonin administered via intraperitoneal injections in mice are also 10 times higher compared with the normal control, which are similar with that in human, providing evidence for the potential clinical value of melatonin in the treatment of male sub(in)fertility due to psychological stress. However, more evidence is needed to prove whether melatonin at such concentration could pose similar effects to testicular cells in human suffering from psychological stress.

In conclusion, our findings demonstrate that melatonin mitigates the restraint stress-induced spermatogenic damage via reducing apoptosis and oxidative stress in testicular cells. It also elucidates that the beneficial effects of melatonin potentially rely on the regulation of NF-κB/iNOS and Nrf2/HO-1 signaling pathway. The present study provides new insights into psychological stress-mediated impairment of spermatogenesis and provides evidence for protective role of melatonin in male fertility. The prospective underlying mechanisms suggest that antioxidants such as melatonin treatment might be a putative strategy to ameliorate the decline in semen caused by psychological stress, with the potential value serve as tools for clinical purpose for male fertility preservation.

## Materials and Methods

### Animal experiments

Male BALB/c 6-week-old mice were purchased from Shanghai SLAC Laboratory Animal Co., Ltd., and were housed at the Department of Animal Experiments, Medical School of Shanghai Jiao Tong University. The mice were housed at 25 °C under controlled conditions (lights on from 8:00 AM to 6:00 PM). In total, 40 mice were randomly assigned to the following groups: control (treated with vehicle without stress), control+M (treated with melatonin without stress), stress (treated with vehicle and stress), and stress+M (treated with melatonin and stress). Restraint stress was induced as described previously^16, 17^. Briefly, the control mice were categorized into groups and allowed contact with each other. Conversely, the stress mice (7-week-old) were individually subjected to 6 h/day of immobilization stress in 50 mL conical centrifuge tubes (Corning Life Sciences, Tewksbury MA, USA) for 35 days between 9:00 AM and 03:00 PM. Spermatogenesis requires approximately 35 days to produce spermatozoa from spermatogonial stem cells (SSCs) in mice^70^. Hence, the mice were treated for 35 days in the current study. The tubes were typical of multiple punctures that allowed for a close fit to the male mice and maintained sufficient ventilation. During this period, all mice were deprived of water and food to diminish the confounding factors. Melatonin was dissolved in 1% ethanol (in normal saline). For melatonin (M) treatment, control+M, and stress+M group mice were administered intraperitoneal injections of M (10 mg/kg/day, Sigma-Aldrich, St. Louis, MO, USA) before the period of restraint stress every day (9:00 AM) for 35 days while the control and stress group mice were injected with an equal volume of vehicle, a dosage which effectively modulates neuroinflammation by decreasing NF-κB activation cascade and oxidative stress by increasing Nrf2 expression experimental diabetic mice and ameliorates busulfan-induced spermatogonial stem cell oxidative apoptosis in mouse testes^32, 43^. The body weight and food intake of the animals in each group were recorded weekly. All mice were euthanized after anesthesia by inhaling isoflurane (RWD Life Science Co., Ltd, Shenzhen, China). Serum, testes and cauda epididymis were harvested for further analysis.

All animal procedures were approved by the Institutional Animal Care and Use Committee of Shanghai and performed in accordance with the National Research Council Guide for Care and Use of Laboratory Animals. Efforts were made to minimize the suffering of the animals and limit the number of animals used in the study.

### Sperm isolation

Sperm was obtained as described previously^17^. The method was slightly modified, Cauda epididymis was dissected from the sacrificed mice, punctured, and incubated for 30 min in M2 media (Sigma-Aldrich, St. Louis, MO, USA) at 37 °C. The supernatant was removed to a new tube, centrifuged (3000 × *g* for 5 min), washed, and suspended in PBS. Sperm concentration was evaluated under microscopy by using a hemocytometer.

### Hormone assays, melatonin and cytokine analysis

The blood samples withdrawn from all mice were maintained at room temperature for 30 min and centrifuged (3000 rpm, 15 min) to separate the serum, which was preserved at −80 °C. The testes samples were checked weight after cutting. 10 times volume of PBS were added to the samples and were homogenized, and then centrifuged (3000 rpm, 15 min) to remove the supernatant for the following melatonin analysis. The serum corticosterone level was measured by ELISA analysis using a commercially available kit (R&D Systems, Minneapolis, MN, USA). The minimum detectable dose (MDD) of corticosterone is 0.028 ng/ml and the percent coefficient of variation (%CV) < 8%. TNF-α were also assayed by the ELISA kit (CUSABIO, Wuhan, China). MDD of TNF-α is 15.6 pg/ml and %CV < 8%. Melatonin was measured by the ELISA kit (Beijing Baiaolaibo Science and Technology Co., Ltd, Beijing, China). MDD of melatonin is 2 ng/L and %CV < 8%.

### Testicular histology

Testicular biopsies from mice were fixed overnight in Bouin’s fixative, embedded in paraffin, and sectioned into 4-μm thick slices. The sections were stained with hematoxylin and eosin (H&E) and spermatogenesis was assessed under a microscope.

### Terminal deoxynucleotidyl transferase-mediated dUTP-biotin nick end labeling (TUNEL) assay

To detect apoptosis, paraffin-embedded sections and cultured testicular cells were stained with the TUNEL assay using an *in situ* apoptosis detection kit (Roche, Mannheim, Germany) according to the manufacturer’s protocols. DAPI was used to counterstain the nuclei. DNase I treatment was used as a positive control for the assay. To assess the apoptosis of testicular cells, 100 seminiferous tubules were observed in different sections by microscopy. The seminiferous tubules with more than 6 TUNEL-positive cells were counted and compared with the total number of seminiferous tubules to yield a percentage of tubules with a positive TUNEL response^22^. For testicular cells *in vitro* experiment, 10 different visions were observed and TUNEL-positive cells were counted to calculate the ratio of apoptotic cells in each group.

### Determination of oxidative stress in testes tissue

2′,7′-Dichlorodihydrofluorescein diacetate (DCFA-DA) staining was applied to detect the ROS levels of testes according to the manufacturer’s protocol (Yeasen Biotech Co., Ltd., Shanghai, China). Briefly, fresh testicular cells were acquired by enzymatic digestion comprising of 4 mg/mL collagenase IV (Gibco, Carlsbad, CA, USA), 2.5 mg/mL hyaluronidase (Sigma, Louis, MO, USA), 2 mg/mL trypsin (Sigma, Louis, MO, USA), and 1 μg/μl DNase I (Roche, Mannheim, Germany) as described previously^71^. The same number (1 × 10^6^) of testicular cells was counted by using a hemocytometer and incubated with DCFA-DA at 37 °C for 25 min. The fluorescence intensity was utilized to analyze the level of ROS. Testicular SOD (Nanjing Jiancheng Bioengineering Institute, Nanjing, China) and GSH (Nanjing Jiancheng Bioengineering Institute, Nanjing, China) were measured with respective kits according to the manufacturer’s instructions. MDD of SOD is 0.5 U/ml and %CV < 6%. MDD of GSH is 0.3 mg/L and %CV < 5%.

### H_2_O_2_ and melatonin treatment

As described above, fresh testicular cells were isolated. 5 × 10^5^ cells were seeded in 6-well plates and then were treated with 100 μM and 50 μM H_2_O_2_ for 1 h. All the cells were collected for TUNEL staining 24 h later. According to the results of TUNEL assay, 50 μM H_2_O_2_ were chosen to co-treat testicular cells with melatonin. According to the concentrations of melatonin in testes of mice treated with melatonin, 50 μM H_2_O_2_, both 50 μM H_2_O_2_ and 2500 ng/L melatonin, and both 50 μM H_2_O_2_ and 1000 ng/L melatonin were selected to treat testicular cells *in vitro* at 37 °C in 5% CO2 overnight.

### Western blotting

The testes and testicular cells were lysed in cold RIPA buffer (Beyotime Institute of Biotechnology, Hangzhou, China) supplemented with protease inhibitor cocktail (TransGen Biotech, Beijing, China) and phosphatase inhibitor (Transgen Biotech, Beijing, China)) at 1:100 for 30 min on ice; the cell lysates were cleared by centrifugation at 12,000 × *g* for 10 min. The nuclear protein used for analysis of p65 was extracted according to the Nucleoprotein Extraction Kit (Sangon Biotech, Shanghai, China), and the concentration was measured by the BCA kit (Thermo Scientific, Rockford, IL, USA). An equivalent amount of the proteins (20 μg) was loaded and separated on 10% SDS-polyacrylamide gel and transferred to PVDF membranes (Millipore, Billerica, MA, USA). The membranes were blocked with 5% non-fat milk and probed with specific primary antibodies including anti-ACTB at 1:5000 (Proteintech Group,Inc., Chicago, USA), anti-Heme Oxygenase-1 at 1:500 (HO-1, Santa Cruz, CA, USA), anti- nuclear factor erythroid 2-related factor 2 at 1:500 (Nrf2, Wanleibio), anti- Bcl-2-associated X protein at 1:1000 (BAX, Abcam, Cambridge, UK), anti- B-cell lymphoma-2 at 1:1000 (Bcl2, Abcam, Cambridge, UK), anti-cleaved Caspase3 at 1:1000 (Cell Signaling Technolgy, CST, Beverly, MA, USA), anti-phos-IkBα at 1:1000 (nuclear factor of kappa light polypeptide gene enhancer in B-cells inhibitor, alpha, CST, Beverly, MA, USA), anti-p65 at 1:1000 (CST, Beverly, MA, USA), and anti-inducible nitric oxide synthase at 1:1000 (iNOS, Proteintech) at 4 °C overnight. After three washes in TBS containing 0.1% Tween-20 (TBST) for 10 min each, the membranes were incubated with the appropriate goat anti-rabbit IgG antibody for 2 h at room temperature. The membranes were washed three times for 10 min with TBST, and the signals were measured using an enhanced chemiluminescence (ECL) detection kit (NCM Biotech, Suzhou, China). The integrated density values were calculated by comparing the signals of target proteins to that of the housekeeping ACTB. The immunoreactive band intensities in Western blotting were quantified by ImageJ software (NIH).

### Statistical analysis

All the values were presented as mean ± SEM. We tested for normality of all data by Shapiro–Wilk test and homogeneity of variances by Levene test, and then Two-tail Student’s t-test or ordinary One-way analysis of variance (ANOVA) with Tukey’s multiple comparisons test was used to evaluate statistical differences via GraphPad Prism version 6 (GraphPad Sofware). The data between Stress and stress+M group as well as that between H_2_O_2_ and H_2_O_2_+M were analyzed by Two-tail Student’s t-test, with the rest by ANOVA with Tukey’s multiple comparisons test. Differences were considered statistically significant when p-value was <0.05.

### Data Availability

All data generated or analyzed during this study are included in this published article (and its Supplementary Information files).

## Electronic supplementary material

Supplementary information
