# Supplementary material for: Melatonin ameliorates restraint stress-induced oxidative stress and apoptosis in testicular cells via NF-κB/iNOS and Nrf2/ HO-1 signaling pathway
Source: Sci Rep. 2017 Aug 29;7:9599. doi: 10.1038/s41598-017-09943-2 (PMC5575312; doi:10.1038/s41598-017-09943-2)
Supplement: Supplementary file 1 — Supplementary information [file 41598_2017_9943_MOESM1_ESM.pdf]

Melatonin ameliorates restraint stress-induced oxidative stress and apoptosis in testicular cells via NF-κB/iNOS and Nrf2/ HO-1 signaling pathway

Ying Guo, Junyan Sun, Ting Li, Qiuwan Zhang, Shixia Bu, Qian Wang, Dongmei Lai*

International Peace Maternity and Child Health Hospital, School of Medicine, Shanghai Jiao Tong University, Shanghai, 200030, China

***Correspondence information:** Professor Dongmei Lai, The International Peace Maternity and Child Health Hospital, School of Medicine, Shanghai Jiaotong University, Shanghai 200030, China. Tel：86-21-64070434; Fax：86-21-64074642; E-mail：laidongmei@hotmail.com.

Supplemental Fig. 1

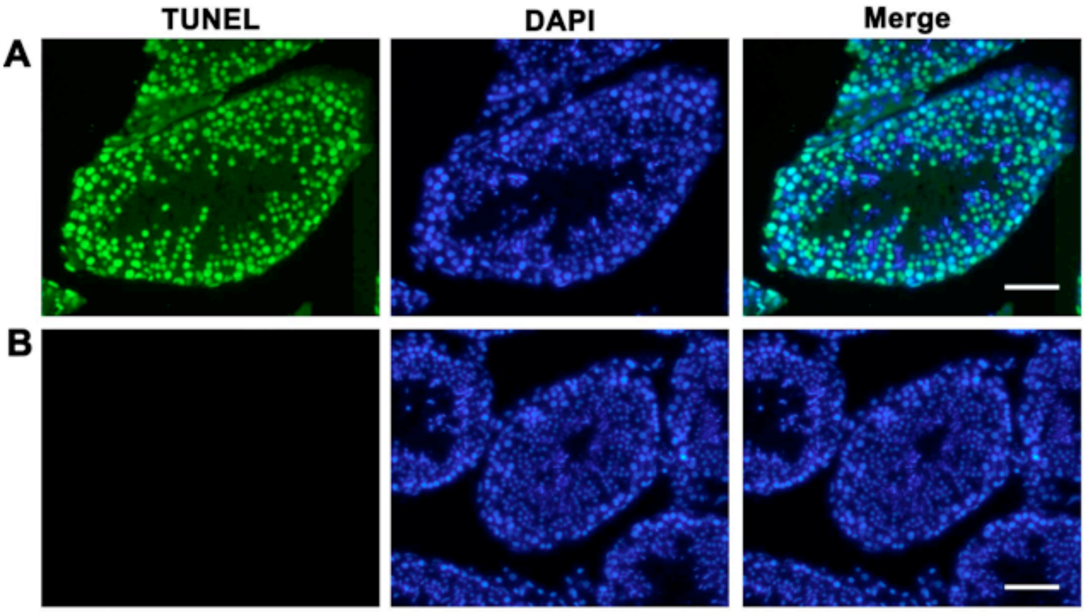

Supplemental Fig. 1 Control of TUNEL assay. (A) DNase I treatment was used for positive control for TUNEL assay. (B) Staining solution in

the kit was chosen as the negative control for TUNEL assay. Bar=100 μm.

Supplemental Fig. 2

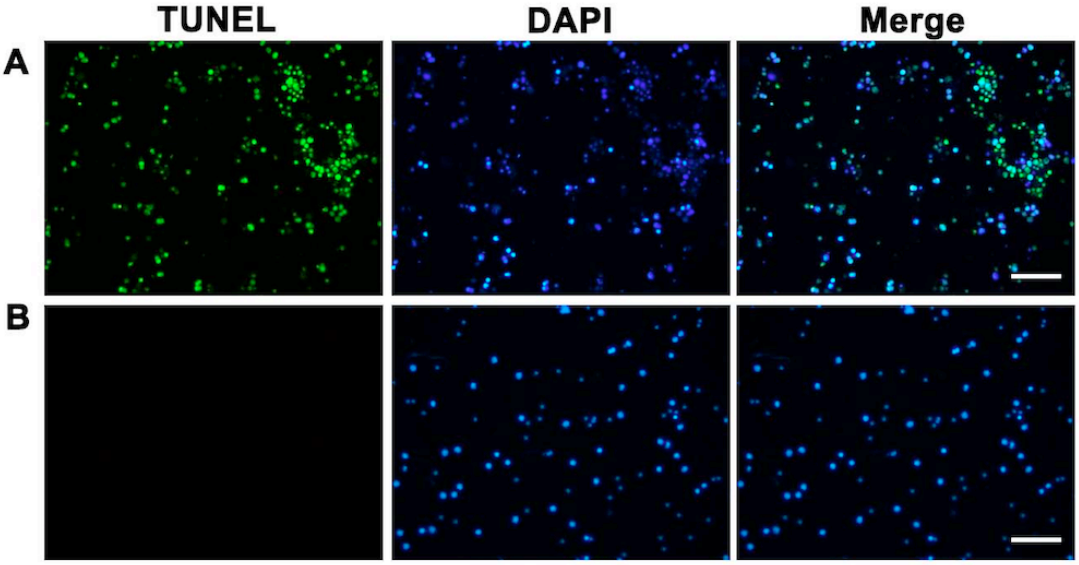

Supplemental Fig. 2 Control of TUNEL assay of testicular cells. (A) DNase I treatment was used for positive control for TUNEL assay. (B) Staining solution in the kit was chosen as the negative control for TUNEL assay. Bar=100 μm.

Supplemental Table 1

Melatonin Concentration in serum and testes

Concentration of melatonin (ng/L)								
	Serum				Testes			
	1h	2h	4h	6h	1h	2h	4h	6h
Control	21.0<br>± 1.0	14.7<br>± 0.3	16.3<br>± 0.7	15.6<br>± 0.4	233.3<br>± 8.8	145.0<br>± 2.9	164.0<br>± 4.6	145.5<br>± 8.7
Control+M	256.7<br>± 17.6	159.0 ±<br>10.5	97.3<br>± 13.9	16.1<br>± 1.3	2223.0<br>± 48.1	1669.0<br>± 10.7	977.3<br>±119.8	147.0<br>± 6.2
Stress	19.7<br>± 1.7	18.3<br>± 1.3	17.3<br>± 1.2	15.8<br>± 2.1	200.0<br>± 11.5	195.0<br>± 11.0	177.3<br>± 10.3	136.9<br>± 4.6
Stress+M	246.0<br>± 18.5	155.0<br>± 10.7	85.3<br>± 5.8	17.43 ±<br>1.1	2391.0<br>± 177.8	1515.1<br>± 99.2	1154<br>± 129.6	142<br>± 9.3
